# TTI-621 (SIRPαFc), a CD47-blocking cancer immunotherapeutic, triggers phagocytosis of lymphoma cells by multiple polarized macrophage subsets

**DOI:** 10.1371/journal.pone.0187262

**Published:** 2017-10-30

**Authors:** Gloria H. Y. Lin, Vien Chai, Vivian Lee, Karen Dodge, Tran Truong, Mark Wong, Lisa D. Johnson, Emma Linderoth, Xinli Pang, Jeff Winston, Penka S. Petrova, Robert A. Uger, Natasja N. Viller

**Affiliations:** Trillium Therapeutics Inc., Mississauga, ON, Canada; Center for Cancer Research, UNITED STATES

## Abstract

Tumor-associated macrophages (TAMs) are heterogeneous and can adopt a spectrum of activation states between pro-inflammatory and pro-tumorigenic in response to the microenvironment. We have previously shown that TTI-621, a soluble SIRPαFc fusion protein that blocks the CD47 “do-not-eat” signal, promotes tumor cell phagocytosis by IFN-γ-primed macrophages. To assess the impact of CD47 blockade on diverse types of macrophages that are found within the tumor microenvironment, six different polarized human macrophage subsets (M(-), M(IFN-γ), M(IFN-γ+LPS), M(IL-4), M(HAGG+IL-1β), M(IL-10 + TGFβ)) with distinct cell surface markers and cytokine profiles were generated. Blockade of CD47 using TTI-621 significantly increased phagocytosis of lymphoma cells by all macrophage subsets, with M(IFN-γ), M(IFN-γ+LPS) and M(IL-10 + TGFβ) macrophages having the highest phagocytic response. TTI-621-mediated phagocytosis involves macrophage expression of both the low- and high-affinity Fcγ receptors II (CD32) and I (CD64), respectively. Moreover, macrophages with lower phagocytic capabilities (M(-), M(IL-4), M(HAGG+IL-1β)) could readily be re-polarized into highly phagocytic macrophages using various cytokines or TLR agonists. In line with the in vitro study, we further demonstrate that TTI-621 can trigger phagocytosis of tumor cells by diverse subsets of isolated mouse TAMs ex vivo. These data suggest that TTI-621 may be efficacious in triggering the destruction of cancer cells by a diverse population of TAMs found in vivo and support possible combination approaches to augment the activity of CD47 blockade.

## Introduction

Macrophages are an essential component of the innate immune response, and exhibit notable plasticity and diversity. Traditionally, macrophages have been defined as being polarized into two distinct states: the classically activated M1 phenotype and the alternatively activated M2 phenotype. M1 macrophages are typically activated by LPS or IFN-γ, and are cytotoxic effectors that produce pro-inflammatory cytokines and nitric oxide; whereas M2 macrophages are activated by IL-4 and have anti-inflammatory and immunosuppressive properties. However, it is now increasingly being appreciated that the M1/M2 dichotomy is an over-simplification, and that macrophages found in vivo exist along a spectrum of activation states depending on the ontogeny, local tissue environment and stress signals [1, 2].

Cancer is an ideal paradigm of macrophage diversity, as tumor-associated macrophages (TAMs) are immensely diverse in both phenotype and function. TAMs are a major cellular component of many murine and human tumors, and depending on the local milieu, can be anti-tumorigenic (pro-inflammatory and phagocytic towards tumor cells) or pro-tumorigenic (promoting tumor cell survival, metastasis, angiogenesis, as well as suppression of surrounding immune cells) [3, 4]. High macrophage infiltration has been associated with poor patient prognosis as in follicular lymphoma, thyroid and lung cancers [5–7], but high TAM density has also been shown to correlate with increased survival in other malignancies such as pancreatic cancer and colorectal cancer [8, 9], emphasizing the importance of understanding how novel macrophage-directed anti-cancer agents will work on heterogeneous macrophage subsets in vivo.

Although TAMs have the capacity to phagocytose cancer cells that express pro-phagocytic signals, tumor cells often evade macrophage-mediated destruction by increased cell surface expression of CD47, which delivers a “do-not-eat” signal by binding signal-regulatory protein α (SIRPα) on the surface of macrophages [10]. TTI-621 (SIRPαFc) is a novel immunotherapeutic consisting of the CD47-binding domain of human SIRPα linked to the Fc region of human IgG1. It is designed to block the CD47 “do-not-eat” signal and engage macrophage Fcγ receptors to enhance phagocytosis and anti-tumor activity. We have previously shown that TTI-621 triggers tumor cell phagocytosis by M(IFN-γ) macrophages in vitro and inhibits tumor growth in vivo [11]. Given the extraordinary heterogeneity of macrophages in vivo and of TAMs in particular, a crucial question is whether TTI-621 is efficacious in triggering phagocytosis by diverse macrophage subsets. In this study, we have assessed the extent to which TTI-621 can trigger phagocytosis of lymphoma cells by six distinct subsets of polarized human macrophages.

## Materials and methods

### Cell line

The human diffuse large B cell lymphoma (DLBCL) cell line, Toledo (CRL-2631), was purchased from ATCC and was maintained in RPMI/10% FBS.

### SIRPαFc protein

TTI-621 consists of the N-terminal V domain of human SIRPα (GenBank AAH26692) fused to the human IgG1 Fc region (hinge-CH2-CH3, UniProtKB/Swiss-Prot, P01857). The construct was generated by overlapping PCR using standard molecular biology techniques and expressed in stably transfected CHO-S cells (Invitrogen). Proteins were purified from culture supernatant using protein A and hydrophobic interaction chromatography, concentrated, and residual endotoxin removed. Control human IgG1 Fc proteins lacking the SIRPα domain were also generated and similarly purified. All proteins displayed >99% purity by HPLC and <0.4 EU/mg endotoxin.

### Generation of polarized human macrophages

Heparinized whole blood was obtained from normal healthy human donors (Biological Specialty Corporation) and informed consent was obtained from all donors. Biological Specialty Corporation obtains samples from FDA-registered collection centers and thus separate IRB approval is not required. CD14+ monocytes isolated from PBMC were used to generate monocyte-derived macrophages as previously described [11]. One day prior to the phagocytosis assay the monocyte-derived macrophages were either left untreated in M-CSF media (M(-) macrophages) or treated overnight with 20 ng/mL M-CSF and 300 ng/mL interferon-gamma (IFN-γ) (PeproTech) (M(IFN-γ)), 50 ng/mL IFN-γ and 50 ng/mL LPS (MD Biosciences) (M(IFN-γ+LPS)), 20 ng/mL IL-4 (PeproTech) (M(IL-4)), 20 ng/mL IL-1β (PeproTech) and 50 μg/mL heat aggregated IgG (HAGG) (M(HAGG+IL-1β)) or 20 ng/mL IL-10 (PeproTech) and 20 ng/mL TGFβ (PeproTech) (M (M(IL-10 + TGFβ)). Macrophages were harvested using Enzyme-Free Cell Dissociation Buffer (ThermoFisher).

### Generation of heat aggregated IgG

Heat aggregated IgG (HAGG) was obtained by heating human IgG (Sigma) in PBS at a concentration of 5 mg/mL at 63°C for 1 hour. The solution was chilled on ice for 15 minutes followed by centrifugation at 1100g for 15 minutes to remove insoluble aggregates. The concentration of HAGG was determined by measuring the absorbance of the soluble fraction at 280 nm. HAGG was added to the culture medium at concentration of 50 μg/mL.

### Repolarization of polarized human macrophages

One day following polarization of macrophages into M(-), M(IL-4), and M(HAGG+IL-1β), polarization media was washed off, and cells were treated with 20 ng/mL IFN-γ, 20 ng/mL IL-10, 1 μg/mL LPS (MD Biosciences), 1 μg/mL R848 (InvivoGen), 1000 U/mL IFN-α2a (PBL Assay Science), 10 μg/mL Poly (I:C) (InvivoGen), or 10 μg/mL ODN2395 CpG (InvivoGen) overnight. On the following day, macrophages were harvested using enzyme-free cell dissociation buffer (ThermoFisher).

### Phagocytosis assay

The confocal microscopy and flow cytometry based phagocytosis assays were performed as previously described [11]. %Phagocytosis was assessed as the % of live, single, CD14+CD11b+ macrophages that were VPD450+. Representative flow cytometry dot plots and the gating strategy used can be seen in S2 Fig. The flow cytometry-based phagocytosis assay was validated by both confocal microscopy and imaging flow cytometry to ensure that phagocytic events represent engulfment, and not adhesion, of target cells. Moreover, a tumor cell-specific CD19 mAb was added after the phagocytosis assay to confirm that VPD450+ macrophages are CD19-, and thus represent phagocytosis, and not adhesion (S3 Fig).

### Immunophenotyping of macrophages

Macrophages were stained with a Near-IR LIVE/DEAD fixable dead cell stain (Invitrogen) and the following antibodies in three independent cocktails: FITC-conjugated anti-CD16 (Clone CB16, ebioscience), PE-conjugated anti-MerTK (Clone 108928, R&D Systems), APC-conjugated anti-CD206 (Clone 19.2, BD Biosciences), V450-conjugated anti-CD86 (Clone FUN-1, BD Biosciences), FITC-conjugated anti-CD32 (Clone FL18.26, BD Biosciences), PE-conjugated anti-SIRPα (Clone 15–414, AbSerotec), APC-conjugated anti-CD200R (Clone OX-108, Biolegend), eFluor450-conjugated anti-HLA-DR (Clone L243, ebioscience), PE-conjugated anti-CD80 (Clone 2D10, ebioscience), APC-conjugated anti-CD163 (Clone GH1/61, ebioscience), PerCP-conjugated anti-CD14 (Clone MoP9, BD Biosciences) and V450-conjugated anti-CD64 (Clone 10.1, BD Biosciences). Cells were washed and resuspended in stabilizing fixative (BD Biosciences), and data was acquired on a FACSVerse flow cytometer using identical Tube Settings to allow for a comparison of fluorescence values across experiments and donors. Macrophages were identified as live, single cells. Doublets were excluded by SSC-W and SSC-H discrimination. The expression level of each of the markers was expressed as fold changes relative to M(-) or non-repolarized M(-), M(IL-4) and M(HAGG+IL-1β) using GraphPad Prism software.

### Cytokine and chemokine production by six distinctly polarized macrophages

Culture supernatants were collected from polarized macrophage subsets following the overnight polarization treatments. Supernatants were subsequently analyzed for cytokine and chemokine production using BD cytometric beads array kit (BD Biosciences) and LEGENDplex Human Proinflammatory Chemokine Panel (13-Plex) (Biolegend).

### FcγR blockade

Polarized macrophages were individually blocked with 20 μg/mL of anti-CD16 F(ab’)_2_ fragment (Clone 3G8, Ancell), 20 ug/mL of anti-CD32 F(ab’)_2_ fragment (Clone 7.3, Ancell), 20 μg/mL of anti-CD64 F(ab’)_2_ fragment (Clone 10.1, Ancell) or a combination of all three F(ab’)_2_ fragments (20 ug/mL each). Unwashed macrophages were subsequently incubated with a diffuse large B cell lymphoma (DLBCL) cell line (Toledo) in the presence of 1 μM TTI-621 or control Fc protein for two hours. Macrophages were harvested and analyzed by flow cytometry as described.

### Mice and isolation of tumor-associated macrophages (TAMs)

Seven- to nine-week old SHrN hairless NOD.SCID female mice purchased from Envigo (Indianapolis, IN) were inoculated subcutaneously with 10^7^ human DLBCL (Toledo) cells in the right hind flanks. Tumors were excised from euthanized mice when tumor volumes were approximately 1500–2000 mm^3^. Tumor tissues were dissociated and digested into single cell suspensions using the human tumor dissociation kit (Miltenyi Biotec) and gentleMacs tissue dissociator (Mitenyi Biotec) according to the manufacturer’s instructions. CD11b+ cells were subsequently purified using CD11b microbeads and magnetic columns (Mitenyi Biotec). The ex vivo phagocytosis assay was performed with the freshly isolated TAMs as in [11]. CD11b negative tumor cells as well as in vitro expanded tumor cell lines were used as targets.

All mice were maintained under specific pathogen-free conditions in sterile microisolators at the University of Toronto. The animal study was approved by the University of Toronto animal care committee (approved protocol #20011514) in accordance with the regulations of the Canadian Council on animal care.

### Histology analysis of Toledo xenograft tumors

Toledo xenograft tumors were formalin fixed, paraffin embedded and sectioned, followed by staining with hematoxylin and anti-CD31 (polyclonal, Abcam) or anti-F480 (clone A3-1, Abcam) or anti-Mac-2 (Clone M3/38, Cedarlane) antibodies. Stained slides were scanned and subjected to stain separation using a customized python implementation that converts from color space to optical density using an unmixing color vector matrix for the individual stains. The individual isolated stains were then projected into a new color space using MATLAB, and overlaid on the unmixed CD31 image in ImageJ. The histology and image analysis were performed by the STTARR facility under the University Health Network, Toronto.

## Results

### Macrophage subsets vary in expression of surface markers and cytokine production

Macrophages exhibit a high degree of diversity and plasticity in vivo, and it is often unclear to what degree macrophages derived from peripheral blood monocytes in vitro resemble TAMs. In order to assess the activity of SIRPαFc in macrophages of various activation states, we generated six distinct subsets of human monocyte-derived macrophages (MDMs). In accordance with the proposed common framework for macrophage-activation nomenclature [12], monocytes were differentiated into macrophages in the presence of M-CSF and subsequently polarized into M(IFN-γ), M(IFN-γ+LPS), M(IL-4), M(heat-aggregated gamma globulin (HAGG) + IL-1β) or M(IL-10 + TGFβ) subsets. Unpolarized macrophages were denoted as M(-). Macrophage subsets were subjected to extensive immunophenotyping using a panel of myeloid surface markers, and we found that the M(IFN-γ) subset specifically upregulated the high-affinity Fcγ receptor I (CD64), CD80 and CD86, relative to M(-) macrophages, whereas the M(IFN-γ+LPS) upregulated CD80 and CD86, and downregulated CD14, SIRPα and MerTK (summarized in Table 1 and S1 Fig). The co-stimulatory molecules CD80 and CD86 are typically associated with pro-inflammatory M1 macrophages [13].

**Table 1:** Summary of surface markers, cytokines and chemokines expressed by polarized macrophage subsets. Overview of the six different subsets of macrophages that were generated in vitro, and their corresponding expression of cell surface markers (relative to unpolarized (M(-) macrophages), as well as their production of cytokines and chemokines.

Polarized Macrophage	Cell surface phenotype (relative to M(-) Macrophages)	Chemokine and cytokine production
M(IFNγ)	CD64^hi^	CCL-2
	CD80^hi^	CCL-3
		CCL-4
		CCL-5
		CXCL-1
		CXCL-9
		CXCL-10
		CXCL-11
M(IFNγ + LPS)	CD80^hi^	CCL-2
	CD86^hi^	CCL-3
	CD14^lo^	CCL-4
	SIRPα^lo^	CCL-5
	MerTK^lo^	CXCL-1
		CXCL-9
		CXCL-10
		CXCL-11
		IL-12p70
		TNF-α
		IL-6
		IL-1β
M(IL-4)	CD206^hi^	CCL-17
	CD200R^hi^	
M(HAGG + IL-1β)	CD86^lo^	CCL-2
	CD32^lo^	CCL-3
	HLA-DR^lo^	CCL-4
		CCL-5
		CCL-17
		CCL-20
		CXCL-1
		CXCL-5
		TNFα
		IL-6
		IL-8
		IL-10
M(IL-10 + TGFβ)	CD16^hi^	
	CD32^hi^	
	CD163^hi^	
	MerTK^hi^	
	CD14^hi^	
	HLA-DR^lo^	
	CD86^lo^	

M(IL-4) macrophages specifically upregulated CD200R and the mannose receptor CD206, both of which are typically associated with anti-inflammatory M2 macrophages [14]. Interestingly, M(HAGG+IL-1β) macrophages did not upregulate any M2-associated markers, but downregulated the M1 markers CD86 and HLA-DR. Finally, M(IL-10 + TGFβ) macrophages upregulated CD14, the low-affinity FcγR CD16 (FcγRIII), CD32 (FcγRII) as well as the scavenger receptor CD163, and downregulated HLA-DR and CD86 (Table 1).

To further define these polarized macrophages, cell culture supernatants were collected and assessed for cytokine production (Table 1 and S1 Fig). We found that only M(IFN-γ+LPS), macrophages produced IL-12p70, a well-known M1 cytokine [15]. Other pro-inflammatory cytokines, such as TNF-α and IL-6, were produced by both M(IFN-γ+LPS) and M(HAGG+IL-1β) subsets. The immunosuppressive cytokine IL-10 was produced by M(HAGG+IL-1β) macrophages only, whereas TGFβ could be detected by all subsets except M(-). In agreement with previous studies, CCL-17 was found to be expressed at a highest level by the M(IL-4) subset [16], followed by the M(HAGG+IL-1β) subset (Table 1). CXCL-5 (ENA-78) was expressed highly on M(HAGG+IL-1β), followed by the M(IFN-γ) and M(IFN-γ+LPS) subsets. Interestingly, a panel of chemokines was found to be produced similarly by M(IFN-γ), M(IFN-γ+LPS) and M(HAGG+IL-1β) macrophages, including CCL-3 (MIP-1a), CCL-4 (MIP-1b), CXCL-1 (GROa), CCL-5 (Rantes) and CCL-2 (MCP-1) and CXCL-8 (IL-8) and CCL-20 (MIP-3a). In contrast, CXCL-9 (MIG), CXCL-10 (IP-10) and CXCL-11 (I-TAC) were produced by M(IFN-γ) and M(IFN-γ+LPS) macrophages only, but not by the M(HAGG+IL-1β) subset.

These data show that each polarized macrophage subset has a unique and distinct phenotype. Moreover, there is substantial overlap between the subsets in terms of cell surface expression of markers, as well as production of cytokines and chemokines, highlighting that the M1/M2 paradigm of macrophage polarization is an over-simplification.

### TTI-621 triggers phagocytosis of lymphoma cells by all in vitro polarized macrophage subsets

**Fig 1:**
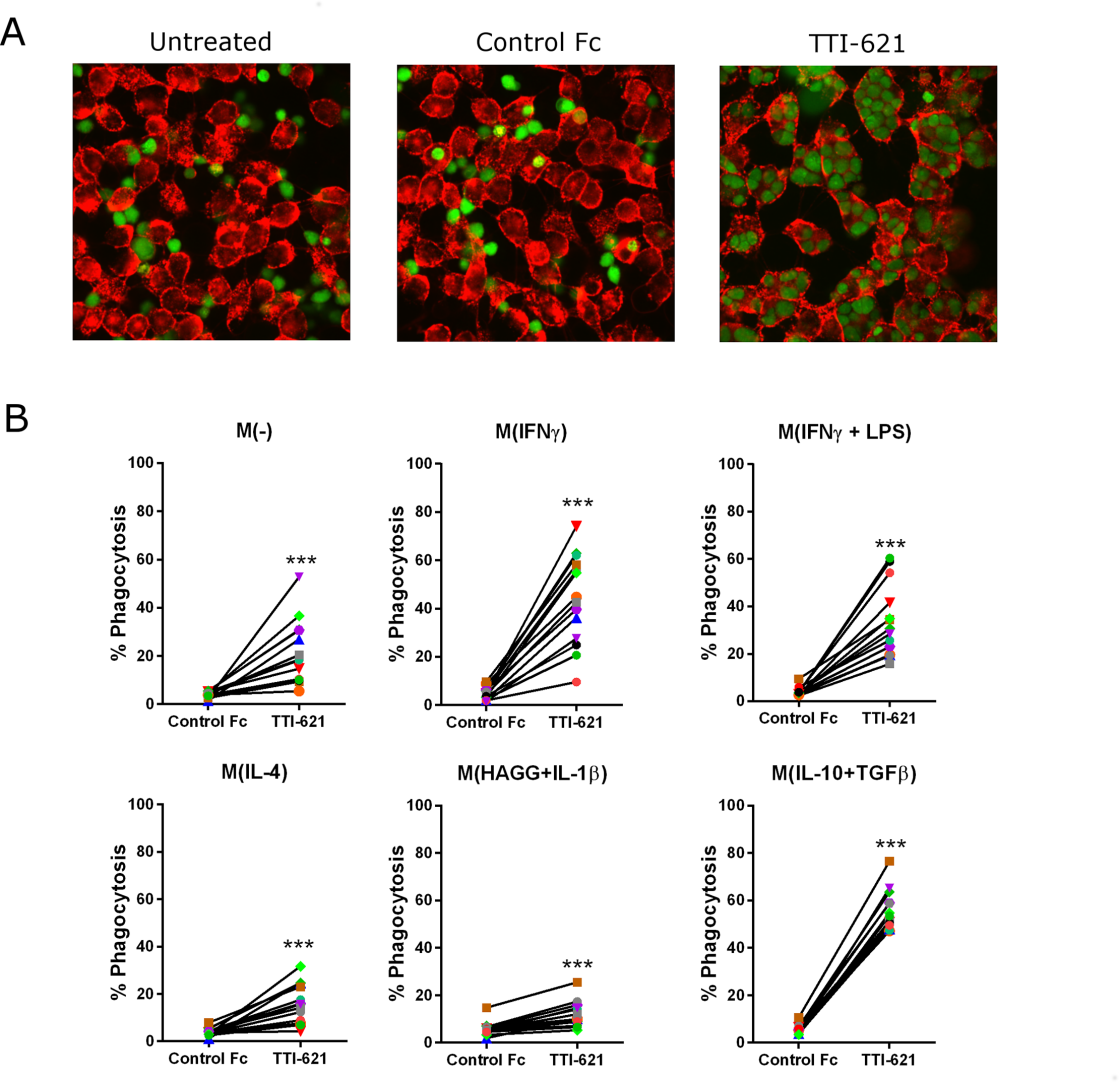
TTI-621 triggers phagocytosis of lymphoma cells by all macrophage subsets. Macrophage subsets were generated as described and co-cultured with DLBCL Toledo cells for two hours in the presence of TTI-621 or isotype-matched control Fc. (A) Phagocytosis was determined by scanning confocal microscopy. Representative images are shown whereby tumor cells and macrophages are stained green and red, respectively. (B) % Phagocytosis was determined by flow cytometry as the % of live, single, CD14+CD11b+ MDMs that were VPD450+. Statistical significance was calculated using a t test where * p ≤ 0.05, ** p ≤ 0.01, *** p ≤ 0.001. Data shown represent n = 14 macrophage donors where each symbol/color represents the same macrophage donor.

We have previously shown that the CD47-blocking agent TTI-621 triggers tumor cell phagocytosis by M(IFN-γ) macrophages in vitro and exhibits anti-tumor activity in vivo [11]. Given the heterogeneity of TAMs, we assessed the ability of TTI-621 to trigger phagocytosis of lymphoma cells by the different macrophage populations, and found that blockade of CD47 using TTI-621 dramatically increased phagocytosis of lymphoma cells by all subsets, relative to cultures treated with control Fc fragment, with M(IFN-γ), M(IFN-γ+LPS) and M(IL-10 + TGFβ) MDMs being superior at phagocytosis (Fig 1 and S2 Fig).

### TTI-621-mediated phagocytosis of lymphoma cells is dependent on macrophage expression of FcγRII (CD32) and FcγRI (CD64)

**Fig 2:**
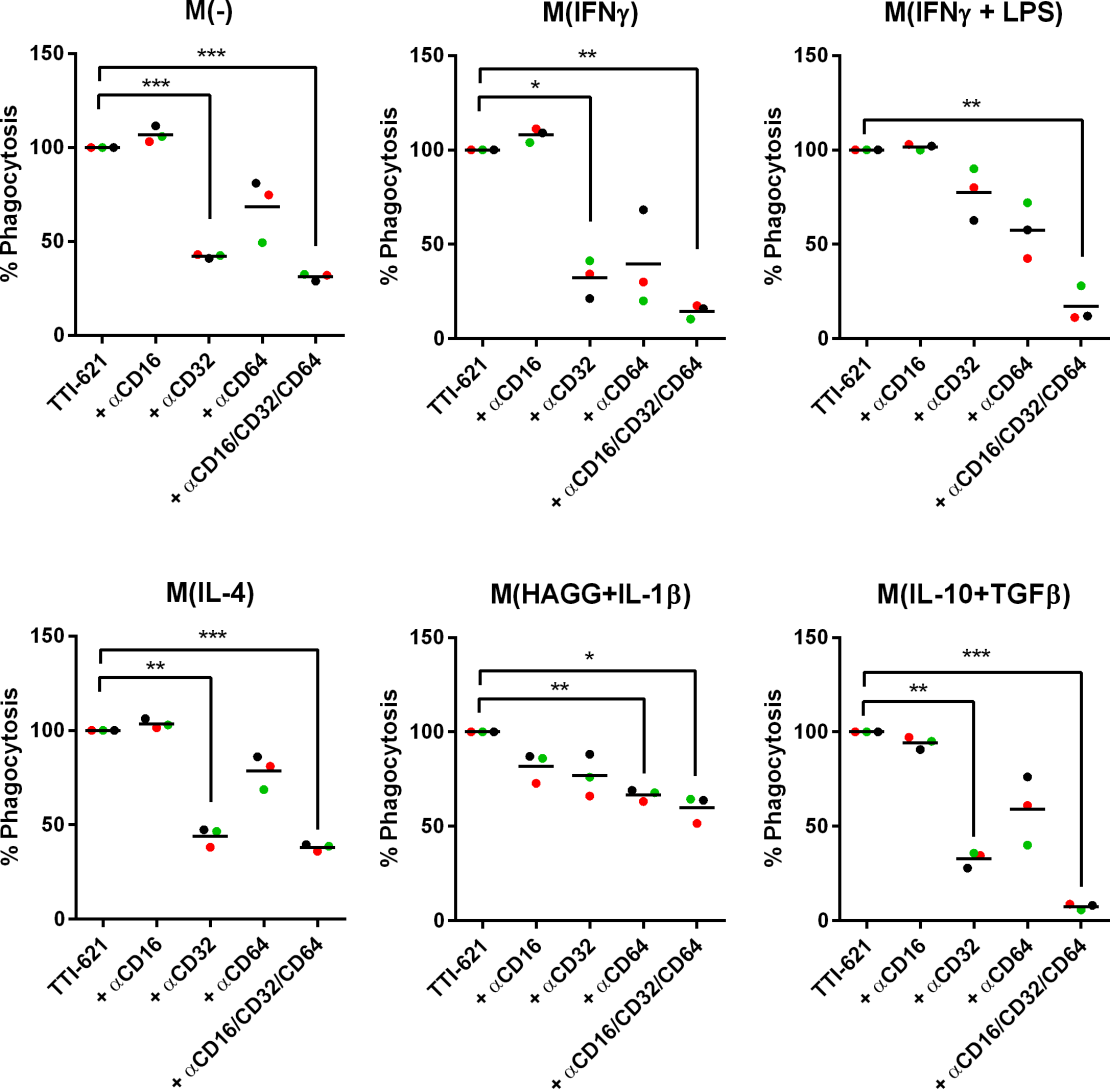
TTI-621-mediated phagocytosis lymphoma cells is dependent on FcγRII (CD32) and FcγRI (CD64). Macrophage subsets were generated as described and co-cultured with Violet Proliferation Dye (VPD450)-labeled DLBCL Toledo cells for two hours in the presence of TTI-621 or isotype-matched control Fc. % Phagocytosis was determined by flow cytometry as the % of live, single, CD14+CD11b+ MDMs that were VPD450+. (B) Blocking F(ab’)_2_ against FcγRs (CD16, CD32 or CD64) were added individually or in combination as indicated during the two-hour phagocytosis assay. Data shown represent n = 3 macrophage donors. Statistical significance was carried out using a one-way ANOVA with Dunnett’s multiple comparisons test.

We next investigated what could be driving this difference in phagocytic capacity between the polarized macrophage subsets in the presence of TTI-621. We found that all macrophage types phagocytosed latex beads to the same extent (data not shown), suggesting that the macrophage subsets with lower phagocytic capabilities (M(-), M(IL-4) and M(HAGG+IL-1β)) do not have an inherent phagocytic defect. Interestingly, the macrophage subsets M(IFN-γ + LPS) and M(IL-10 + TGFβ) that demonstrated highest tumor cell phagocytosis in the presence of TTI-621 were found to expressed highest level of CD64, and CD32/CD16, respectively (S1 Fig). To evaluate the contributions of the FcγRs in TTI-621-mediated tumor cell phagocytosis, individual FcγRs were blocked by anti-CD64, anti-CD32 and anti-CD16 F(ab’)_2_ fragments in a phagocytosis assay. We found that both CD32 and CD64 contribute to TTI-621-mediated phagocytosis by all subsets (Fig 2) whereas CD16 appeared to be dispensable.

### Repolarized M(-), M(IL-4) and M(HAGG+IL-1β) macrophages have an increased phagocytic response to TTI-621

**Fig 3:**
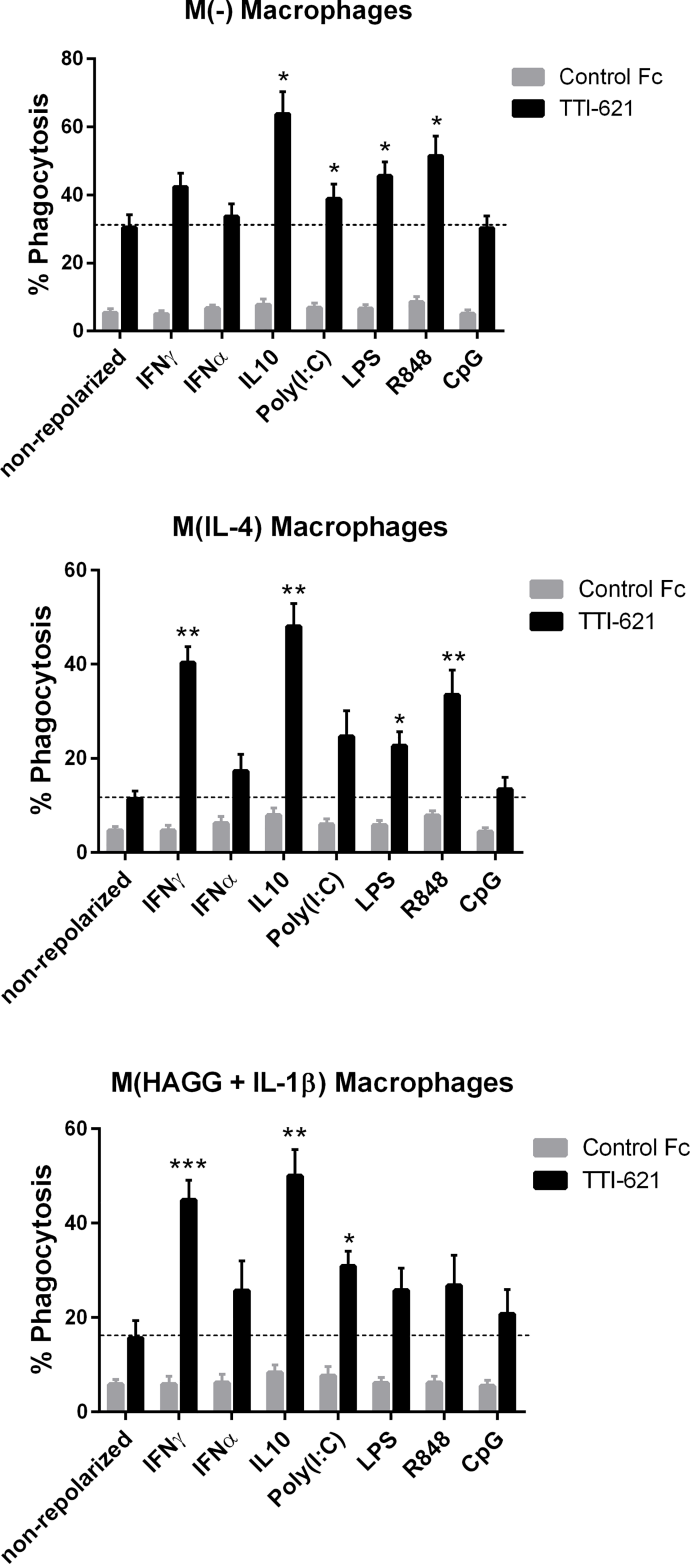
Repolarized M(-), M(IL-4) and M(HAGG+IL-1β) MDMs have an increased phagocytic response to TTI-621. M(-), M(IL-4) and M(HAGG+IL-1β) macrophages were generated as described, and subsequently re-polarized with IFN-γ, IL-10, LPS, R848, IFN-γ, Poly (I:C) or CpG overnight. The resulting repolarized MDM were washed and incubated with Violet Proliferation Dye (VPD450)-labeled DLBCL Toledo cells in the presence of 1 μM TTI-621 or control Fc for two hours. % Phagocytosis was determined by flow cytometry as the % of live, single, CD14+CD11b+ MDMs that were VPD450+. A summary of 4–5 independent experiments is shown. Paired t-test was performed comparing TTI-621 repolarized macrophage subset vs TTI-621 non-repolarized macrophage subset. The dotted lines indicate the phagocytic response of M(-), M(IL-4) and M(HAGG+IL-1β) macrophage in the presence of TTI-621. Statistical significance was calculated using a t test where *p<0.05, **P<0.01 and ***p<0.001.

Although all six types of macrophages showed increased phagocytosis of tumor cells in the presence of TTI-621, M(-), M(IL-4) and M(HAGG+IL-1β) macrophages exhibited slightly lower phagocytic capabilities compared to the M(IFN-γ), M(IFN-γ + LPS) and M(IL-10 + TGFβ) subsets (Fig 1). We evaluated whether the response of M(-), M(IL-4) and M(HAGG+IL-1β) macrophages could be enhanced by repolarizing into an M1-like macrophage state using Type-I and Type-II interferons (IFN-α, IFN-γ) and various TLR agonists (Poly(I:C), LPS, R848 and CpG). We also tested if IL-10, an immunosuppressive cytokine often found within the tumor microenvironment [17], can repolarize macrophages into M(IL-10 + TGFβ)-like macrophages. We observed significant changes in M1 and M2 cell surface marker expressions following overnight repolarization, an indication that they have switched to a more M(IFN-γ+/-LPS) or M(IL-10 + TGFβ)-like phenotype (data not shown). Overall the phagocytic capacity of M(-), M(IL-4) and M(HAGG+IL-1β) macrophages was enhanced following repolarization with IFN-γ, IFN-α, IL-10, Poly (I:C), LPS, R848, but not with CpG, although the sensitivity to each of the repolarizing agents differed among the three macrophage subsets (Fig 3).

### TTI-621 triggers phagocytosis of lymphoma cells by M1-like and M2-like tumor-associated macrophages ex vivo

**Fig 4:**
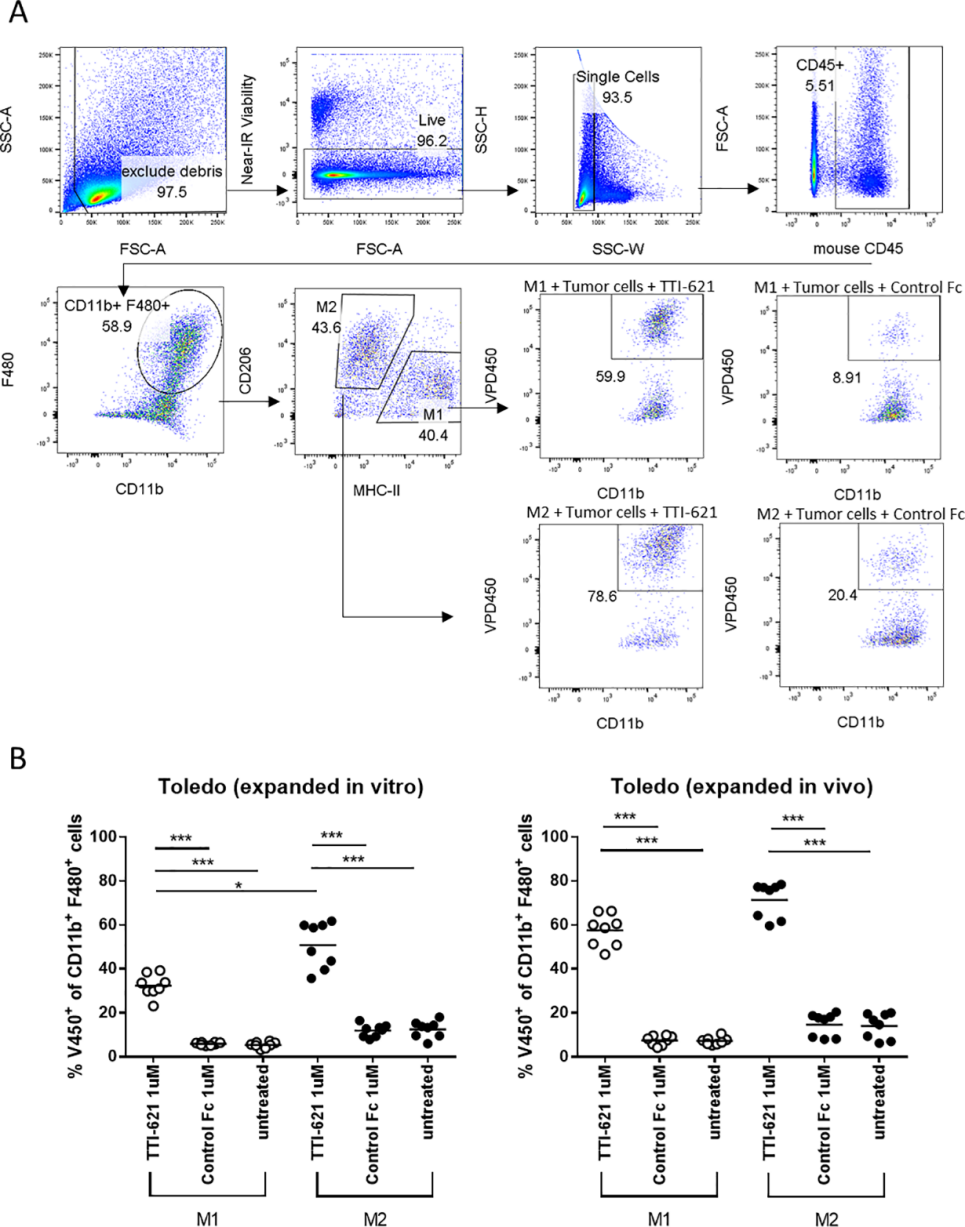
TTI-621 triggers phagocytosis of lymphoma cells by M1-like and M2-like tumor-associated macrophages (TAMs). CD11b+ cells were isolated from DLBCL (Toledo) xenograft tumors as described and co-cultured with Violet Proliferation Dye (VPD450) labeled DLBCL Toledo cells for 2.5 hours in the presence of 1μM TTI-621, isotype-matched control Fc or left untreated. (A) Phagocytosis was assessed by flow cytometry and % phagocytosis was defined as the percentage of macrophages that were VPD450+. TAMs were defined as live, single, CD45+F480+CD11b+ cells and further defined as MHC-II^hi^ CD206^lo^ M1-like and MHC-II^lo^ CD206^hi^ M2-like macrophages. (B) A summary of two independent experiments is shown with human lymphoma target cells that were expanded in vitro (left panel) or purified from excised tumors (right panel). One-way ANOVA with Tukey’s multiple comparisons was performed comparing the % phagocytosis within M1- and M2-like subsets upon various treatments as well as comparing TTI-621-treated M1- and M2-like macrophages towards in vitro or in vivo expanded tumor cell targets. Data shown represent n = 8 mice. Where indicated, *p<0.05 and ***p<0.001.

Given that TTI-621 can trigger phagocytosis of Toledo tumor cells by all six subsets of in vitro polarized human MDM (Fig 1), we next asked whether TTI-621 can trigger phagocytosis of tumor cells by both M1- and M2-like tumor-associated macrophages (TAMs) isolated from a tumor in vivo. To be consistent with our in vitro data, we set up a xenograft in vivo study using the same DLBCL tumor (Toledo) cell line. Based on the histology analysis, the tumor-associated macrophages were found to disperse evenly throughout the Toledo xenograft tumor without preferential localization towards the intravascular or perivascular region (S4 Fig). Tumors were harvested and CD11b+ myeloid cells were isolated, after which a phagocytosis assay was performed ex vivo using TAMs as effectors. TAMs can be divided into two main subsets based on the expression of MHC-II and CD206, where M1-like TAMs are MHC-II^hi^ CD206^lo^, and M2-like TAMs are MHC-II^lo^ CD206^hi^ [18]. By gating on the F480^+^ CD11b^+^ MHC-II^hi^ CD206^lo^ M1-like and F480^+^ CD11b^+^ MHC-II^lo^ CD206^hi^ M2-like populations (Fig 4A), we found that TTI-621 dramatically increased the phagocytosis of in vitro expanded, as well as of ex vivo purified, lymphoma cells compared to control Fc (Fig 4A and 4B). Interestingly, in this model system, M2-like TAMs were found to have an increased phagocytic capability towards in vitro expanded lymphoma targets in response to TTI-621 compared to M1-like TAMs.

## Discussion

Blockade of the CD47-SIRPα pathway using anti-CD47 antibodies, SIRPαFc or by CD47 siRNA knockdown have been shown to increase phagocytosis of tumor cells by in vitro generated M1 and M2 macrophages [19], which represent two extremes along a continuum of macrophage activation. TAMs are highly heterogeneous and can adopt a spectrum of activation states, and the proportion of each of the various states varies depending not only on the tumor type, but also on disease progression [2, 13]. In this study, we sought to assess whether blockade of CD47 by SIRPαFc could increase phagocytosis of tumor cells by six phenotypically distinct macrophage subsets derived from human monocytes.

We demonstrated that CD47 blockade with TTI-621 increased phagocytosis of lymphoma cells by all macrophage subsets. Consistent with the in vitro findings, TTI-621 also dramatically increased the phagocytosis of human lymphoma cells by both M1-like and M2-like mouse TAMs isolated from xenograft tumors, suggesting that TTI-621 will be efficacious in triggering phagocytosis by the diverse subsets of TAMs found in vivo.

We further demonstrated that the in vitro generated M(-), M(IL-4) and M(HAGG+IL-1β) macrophages, all of which exhibited slightly lower phagocytic capabilities in response to TTI-621 compared to M(IFN-γ+/-LPS) and M(IL-10 + TGFβ) subsets, could readily be repolarized into highly phagocytic macrophages using cytokines (IFN-γ, IFN-α or IL-10) or TLR agonists (LPS, Poly (I:C) or R848), suggesting possible unique approaches for combination therapy. TLR stimulation has previously been shown to enhance phagocytosis of *S*. *pneumoniae* and cryptococci by murine microglia [20, 21]; as well as enhance macrophage-mediated phagocytosis of opsonized erythrocytes [22]. Interestingly, stimulation through TLR-3, -4, and -7 has previously been reported to synergize with CD47 blockade on tumor cells by increasing secretion and cell-surface exposure of calreticulin on macrophages in a Btk-dependent manner [23].

Differences in the phagocytic capacity of polarized macrophages have been reported previously. One study found that macrophages generated in M-CSF phagocytosed more opsonized tumor cells compared to macrophages generated using GM-CSF, IFN-γ and LPS [24]. Furthermore, IL-10-treated macrophages have been shown to have increased phagocytic function [24, 25]. However, other studies have demonstrated that IL-4 -polarized macrophages are less phagocytic [24, 26]. Furthermore, M(GM-CSF) were found to be more phagocytic than M(IL-4 + IL-13) towards primary human glioblastoma cells in the context of CD47 blockade ex vivo [27]. To get a better overview of how various stimuli affect the phagocytic capacity of macrophages, we generated six distinct types of macrophages in vitro. We found that both M(IFN-γ+/-LPS) and M(IL-10 + TGFβ) macrophages had a high phagocytic capacity in response to TTI-621 compared to M(-), M(IL-4) and M(HAGG + IL-1β) macrophages.

We have previously reported that the potent effects of TTI-621 were attenuated when the IgG1 Fc tail of the fusion protein was substituted with an IgG4 Fc region [11]. We extend this finding in the current study by blocking individual FcγRs (CD64, CD32, and CD16) using F(ab’)_2_, and report that both CD64 and CD32, but not CD16, contributed in TTI-621 mediated phagocytosis by all 6 macrophage subtypes. IgG4 Fc binds strongly to CD64 but has weaker interactions with CD32 and CD16 than IgG1 [28]. This suggests that an IgG1 Fc region is necessary for SIRPαFc’s enhancement of phagocytosis by both all macrophage subsets in vivo, and one can speculate that an IgG1 tail will thus be more efficacious across the plethora of phenotypically diverse macrophages found in vivo.

Consistent with the result reported by Nagelkerke et al. [29], we found that CD64 blockade by clone 10.1 F(ab’)_2_ was not as efficient compared with the monoclonal antibody counterpart, whereas the efficiency of blocking FcγRs using F(ab’)_2_ versus mAbs was similar for CD32 and CD16 (data not shown). Although clone 10.1 interferes with IgG binding, the epitope recognized by clone 10.1 is not located at the IgG-binding site [30], therefore, the usage of F(ab’)_2_ likely under-represents the role of CD64 due to a lower capacity for CD64 blockade. On the contrary, data using the intact 10.1 antibody is likely to over-represent the role of CD64 due to the ability of the Fc portion to bind to other FcγRs [31].

Our finding that CD64 plays an important role in TTI-621-mediated phagocytosis conflicts with the postulation that the high-affinity nature of FcγRI results in receptor saturation in the presence of serum IgG, subsequently inhibiting binding of immune complexes and/or opsonized target cells. Interestingly, the high concentration of IgG in serum has been shown to be theoretically sufficient to nearly saturate even low-affinity FcγRs [32]. However, it has been hypothesized that local cytokine production can cause de novo synthesis of free FcγRI; indeed, cytokine-mediated upregulation of FcγRI occurs rapidly and is well documented [33]. Moreover, it has been shown that FcγRI ligand-binding can be regulated inside-out by cytokine activation, leading to efficient competition between immune complexes and pre-bound monomeric IgG [34]. Interestingly, this increase in ligand binding is independent of changes in receptor expression. Numerous mouse studies have also documented critical roles for FcγRI in antibody-mediated treatment in vivo, such as in the B16F10/TA99 melanoma model [35]. These data suggest that local cytokine-mediated FcγRI upregulation, as well as inside-out signaling, may overcome competition by serum IgG during recognition of opsonized target cells in vivo.

This study is the first to demonstrate that CD47 blockade using SIRPαFc triggers phagocytosis of tumor cells by a broad spectrum of human macrophages as well as mouse TAMs isolated ex vivo. We also show that FcγRs CD64 and CD32 contribute to the enhanced phagocytosis mediated by TTI-621 and that macrophage subsets (M(-), M(IL-4) and M(HAGG+IL-1β)) having lower phagocytic capabilities in response to TTI-621 can be converted into highly phagocytic macrophages using cytokines (IFN-γ, IFN-α or IL-10) or TLR agonists (LPS, Poly (I:C) or R848). This study also provides the rationale for combining TTI-621 with TLR agonists in the clinical setting. These results support the ongoing clinical evaluation of TTI-621 in relapsed/refractory hematological malignancies (NCT02663518) and solid tumors and mycosis fungoides (NCT02890368).

## Supporting information

S1 Fig Immunophenotying of six distinctly polarized monocyte-derived macrophages. Six distinctly polarized monocyte-derived macrophages (MDM) were generated as described. Following overnight polarization, macrophages were harvested and stained with a viability dye and antibodies against M1, M2 markers, as well as FcγRs followed by flow cytometry analysis. The expression level of each of the markers were expressed as fold changes relative to M0. Macrophages culture supernatant was harvested for cytokine and chemokine analysis using the BD cytometric beads array and LEGENDplex chemokine array, respectively. Each color represents macrophage subsets that were derived from an independent donor.
(TIF)

S2 Fig Representative dot plots and gating strategy for flow cytometry-based phagocytosis assay. Monocyte-derived macrophages (MDMs) were generated from peripheral blood monocytes of healthy donors as described. Macrophages were co-cultured with Violet Proliferation Dye (VPD450)-labeled tumor cells for two hours in the presence of 1 μM TTI-621 or Control Fc. Phagocytosis was assessed by flow cytometry and % phagocytosis was defined as the percentage of macrophages that were VPD450+. Macrophages were defined as live, single, CD14+CD11b+ cells.
(TIF)

S3 Fig The addition of a tumor-cell specific marker after the phagocytosis assay rules out adhesion. (A) CD19 is highly expressed on Toledo cells (solid black histogram), relative to isotype control (grey shaded histogram). (B) and (C) Monocyte-derived macrophages (MDMs) were generated from peripheral blood monocytes of healthy donors as described. Macrophages were co-cultured with Violet Proliferation Dye (VPD450)-labeled tumor cells for two hours in the presence of 1 μM TTI-621 or Control Fc. Phagocytosis was assessed by flow cytometry and % phagocytosis was defined as the percentage of macrophages that were VPD450+. Macrophages were also stained for CD19 to rule out adhesion to target cells.
(TIFF)

S4 Fig Histology analysis of Toledo xenograft tumors. Single stains were performed on serial sections of Toledo xenograft tumors using antibodies against CD31 (endothelial marker, left panels) and (A) anti-F480 or (B) Mac-2 (macrophage markers, middle panels). Stained slides were subjected to stain separation using a customized python implantation, followed by overlaying on the CD31 image (right panels) to demonstrate localization of the tumor associated macrophages relative to the intratumoral vasculature.
(TIFF)
