# Supplementary figures and images for: TTI-621 (SIRPαFc), a CD47-blocking cancer immunotherapeutic, triggers phagocytosis of lymphoma cells by multiple polarized macrophage subsets
Source: PLoS One. 2017 Oct 30;12(10):e0187262. doi: 10.1371/journal.pone.0187262 (PMC5662218; doi:10.1371/journal.pone.0187262)

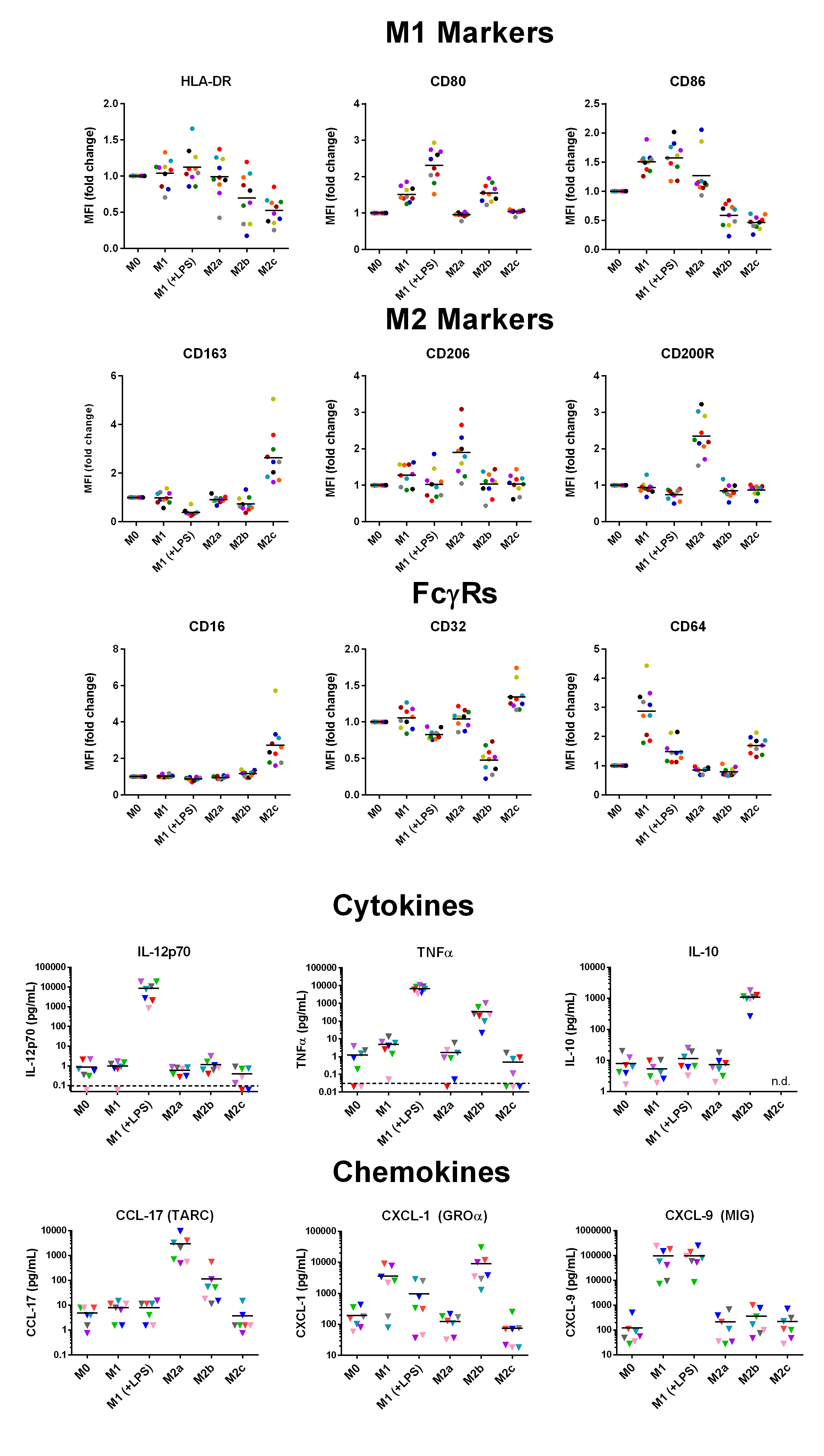

Supplement: S1 Fig — Six distinctly polarized monocyte-derived macrophages (MDM) were generated as described. Following overnight polarization, macrophages were harvested and stained with a viability dye and antibodies against M1, M2 markers, as well as FcγRs followed by flow cytometry analysis. The expression level of each of the markers were expressed as fold changes relative to M0. Macrophages culture supernatant was harvested for cytokine and chemokine analysis using the BD cytometric beads array and LEGENDplex chemokine array, respectively. Each color represents macrophage subsets that were derived from an independent donor. (TIF) [file pone.0187262.s001.tif]

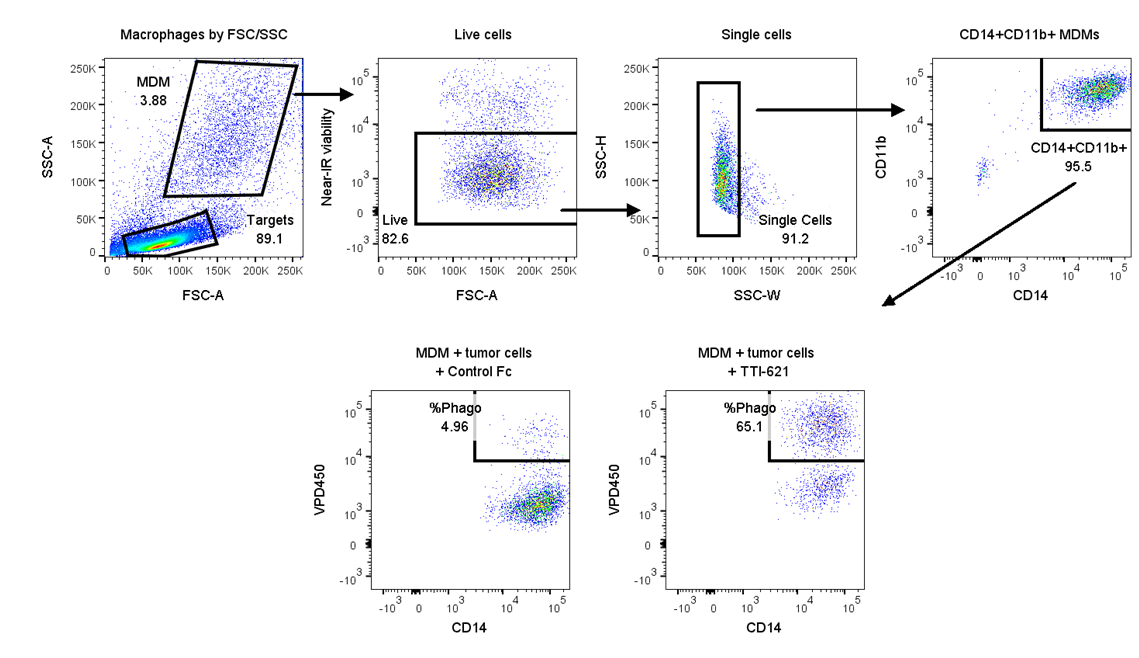

Supplement: S2 Fig — Monocyte-derived macrophages (MDMs) were generated from peripheral blood monocytes of healthy donors as described. Macrophages were co-cultured with Violet Proliferation Dye (VPD450)-labeled tumor cells for two hours in the presence of 1 μM TTI-621 or Control Fc. Phagocytosis was assessed by flow cytometry and % phagocytosis was defined as the percentage of macrophages that were VPD450+. Macrophages were defined as live, single, CD14+CD11b+ cells. (TIF) [file pone.0187262.s002.tif]

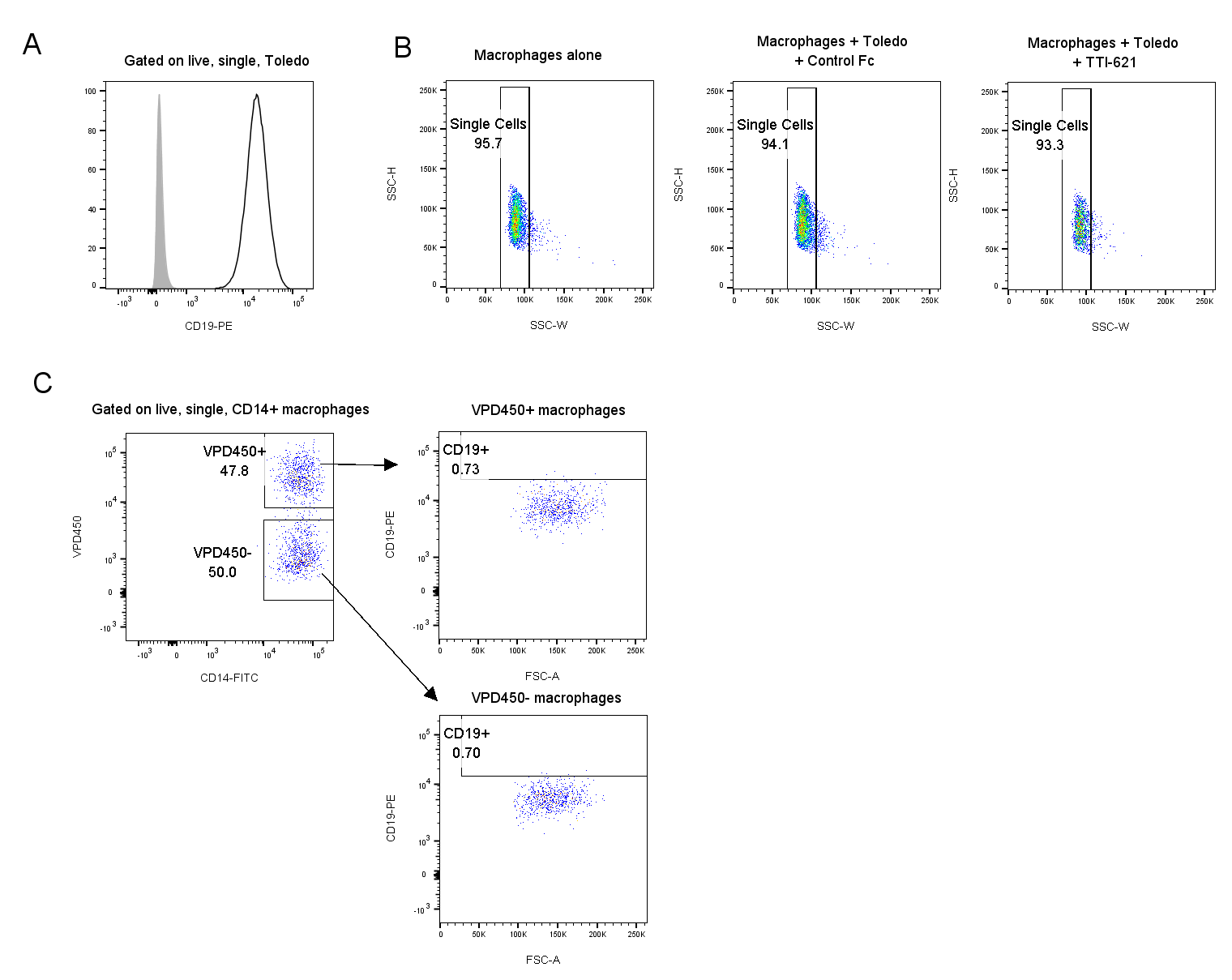

Supplement: S3 Fig — (A) CD19 is highly expressed on Toledo cells (solid black histogram), relative to isotype control (grey shaded histogram). (B) and (C) Monocyte-derived macrophages (MDMs) were generated from peripheral blood monocytes of healthy donors as described. Macrophages were co-cultured with Violet Proliferation Dye (VPD450)-labeled tumor cells for two hours in the presence of 1 μM TTI-621 or Control Fc. Phagocytosis was assessed by flow cytometry and % phagocytosis was defined as the percentage of macrophages that were VPD450+. Macrophages were also stained for CD19 to rule out adhesion to target cells. (TIFF) [file pone.0187262.s003.tiff]

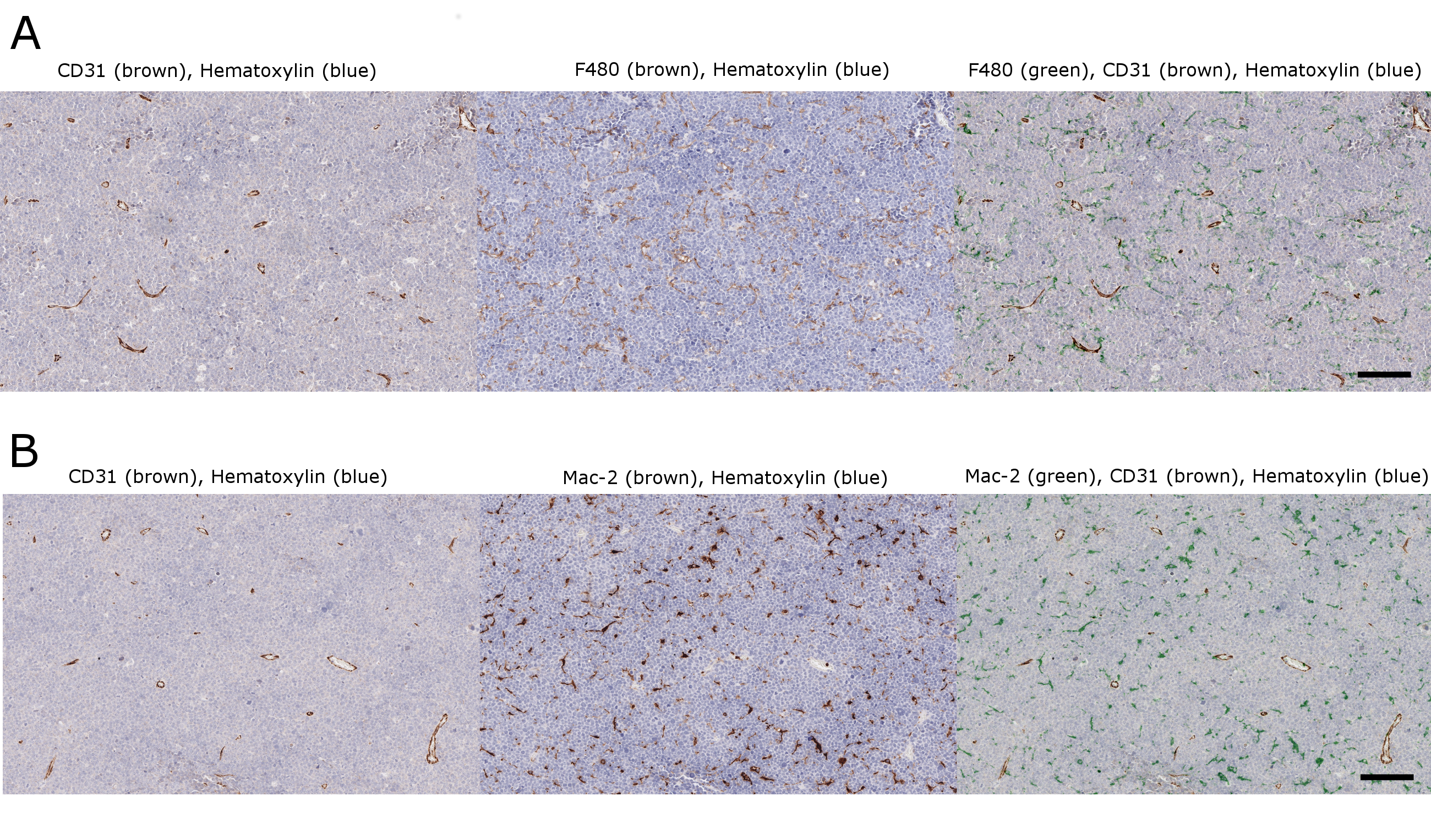

Supplement: S4 Fig — Single stains were performed on serial sections of Toledo xenograft tumors using antibodies against CD31 (endothelial marker, left panels) and (A) anti-F480 or (B) Mac-2 (macrophage markers, middle panels). Stained slides were subjected to stain separation using a customized python implantation, followed by overlaying on the CD31 image (right panels) to demonstrate localization of the tumor associated macrophages relative to the intratumoral vasculature. (TIFF) [file pone.0187262.s004.tiff]
